# Comparison of Procleix Ultrio Elite and Procleix Ultrio NAT Assays for Screening of Transfusion Transmitted Infections among Blood Donors in India

**DOI:** 10.1155/2016/2543156

**Published:** 2016-01-19

**Authors:** Rahul Chaurasia, Diptiranjan Rout, Shamsuz Zaman, Kabita Chatterjee, Hem Chandra Pandey, Abhishek Kumar Maurya

**Affiliations:** ^1^Department of Transfusion Medicine, All India Institute of Medical Sciences, New Delhi 110029, India; ^2^Department of Transfusion Medicine, Sanjay Gandhi Postgraduate Institute of Medical Sciences, Raebareilly Road, Lucknow 226014, India; ^3^Hemogenomics Pvt. Ltd., New Delhi 110044, India

## Abstract

*Background*. Introduction of nucleic acid testing (NAT) has helped in decreasing window period donations, resulting in increased safety of blood supplies. NAT combines the advantages of direct and highly sequence-specific detection of viral genomes. We analysed the performance of newer Procleix Ultrio Elite (PUE) and Procleix Ultrio assay (PUA) for the screening of the viral markers in our donor population.* Material and Methods*. 10,015 donor samples were screened by routine immunoassays and both versions of NAT. NAT yields detected were subjected to viral load estimation and to other serological markers.* Results*. A total of 21 NAT yields were detected; three were positive by both NAT systems, whereas 18 samples were reactive by PUE only. NAT yields include 18 HBV and 3 HCV yields, of which 17 HBV yields were occult infections and 1 was window period (WP) infection. All 3 HCV yields were WP infections. No HIV-1/HIV-2 yield was found.* Conclusion*. Efficient target capture chemistry in the new TMA assay version significantly improved sensitivity. NAT is superior to serological immunoassays for screening of the viral markers; and the efficient target capture system in the newer TMA assay, namely, the PUE system, has significantly improved sensitivity over the earlier versions.

## 1. Introduction

Blood transfusion is safer than ever before through continuous improvements in donor recruitment, meticulous screening, testing of donated blood with increasingly sensitive assays, and appropriate clinical use of blood [1]. The technological advancements at the molecular level TTI screening in the field of transfusion medicine have significantly curtailed the potential risks of transmissible infections through blood transfusions.

Nucleic acid testing (NAT) is a newer technology, introduced in the field of transfusion medicine for molecular diagnosis and characterisation of viral infections. NAT combines the advantages of direct and highly sequence-specific detection of viral genomes (DNA or RNA) with an analytic sensitivity that is several orders of magnitude greater than that of antigen/antibody/antigen-antibody detection techniques or viral isolation methods. The average window period duration during which immunological assays are unable to detect the anti-HIV-1/anti-HIV-2, anti-HCV, and HBsAg, which is estimated to be between 16, 70, and 45 days for HIV-1, HCV, and HBV, respectively, has been markedly reduced with NAT [2]. Introduction of NAT for screening of blood donors had shortened this “window period,” thus significantly increasing the safety of blood supplies [2].

The prevalence of HBV, HCV, and HIV in developed nations like Australia is reported to be very low to the tune of 7.55, 5.34, and 0.31 per 100,000-donor population, respectively [3]. In contrast, developing countries like India still have high prevalence of HBV, HCV, and HIV in the order of 1.8–4%, 0.4–1.09%, and 0.2–1%, respectively [4–9]. High prevalence coupled with inadequate blood screening continues to thwart the blood supply of the nation. Nucleic acid testing along with routine serology is expected to increase the blood safety in countries like India where prevalence of TTI is high [10].

A new-generation individual donor-nucleic acid testing (ID-NAT) based on the principle of transcription-mediated amplification (TMA) assay, namely, the Procleix Ultrio assay (PUA) (previously, Chiron/Gen-Probe, Emeryville/San Diego, CA, USA; now marketed by Grifols Diagnostic Solutions, Inc.), was first approved in 2006 by FDA for TTI screening of blood donors for the simultaneous detection of HIV-1 RNA and HCV RNA and later on extended for detection of HBV in blood donors. Manufacturers improved upon this version and introduced Ultrio Plus assay claiming enhanced sensitivity and automation (on Tigris platform).

Recently, the Procleix Ultrio Elite (PUE) system (previously, Chiron/Gen-Probe, Emeryville/San Diego, CA, USA; now marketed by Grifols Diagnostic Solutions, Inc.) was launched which has the additional ability for the detection of HIV-2 to meet the regional needs in various developing countries like India. The PUE is a multiplex NAT assay designed to detect HIV-1/HIV-2, HBV, and HCV qualitatively in vitro on the fully automated PANTHER instrument.

We evaluated the PUE on the Fully Automated Procleix Panther system and compared the results with routine serology and the earlier PUA.

## 2. Material and Methods

### 2.1. Study Site

The study was conducted at the Main Blood Bank, All India Institute of Medical Sciences, New Delhi, from January 2014 to October 2014 for a total period of 10 months.

### 2.2. Donor Population

A total of 10,015 routine blood donations at our blood bank were screened for viral markers which were mostly from replacement donors or family donors. Administration of the donor history questionnaire (DHQ) and meticulous screening of all the donors by a trained physician prior to blood donation were used to exclude those with high risks of exposure, thus increasing the chances of the TTI.

### 2.3. Serological Testing

All the blood donation units were screened by ELISA using antigen-antibody combination assay (4th-generation kits), namely, Genscreen Ultra HIV Ag-Ab (BIO-RAD), for detection of both HIV-1 and HIV-2, antigen assays (3rd-generation kits), namely, Hepanostika HBsAg Ultra (Biomérieux), for detection of HBV, and antibody assays (3rd-generation kits), namely, Hepanostika HCV Ultra, for detection of HCV infections.

### 2.4. NAT

**Figure 1:**
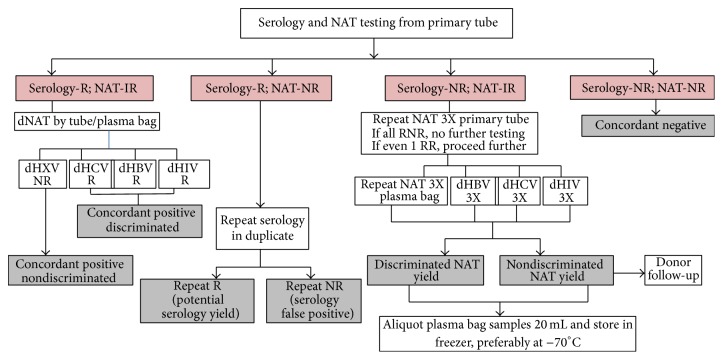
Algorithm for NAT as followed at our centre. R: reactive, IR: initial reactive, NR: nonreactive, RNR: repeat nonreactive, RR: repeat reactive, dNAT: discriminatory NAT, dHXV: discriminatory HCV/HBV/HIV, dHCV: discriminated HCV, dHBV: discriminated HBV, dHIV: discriminated HIV, 1X: tested in singlet, and 3X: tested in triplicate.

ID-NAT was performed in parallel for all the corresponding donor samples using PUE (Automated Panther platform) and PUA (semiautomated platform) for the qualitative detection of the genomic sequences of HCV, HBV, HIV-1, and HIV-2 (only in case of PUE and not in PUA). The principle of the TMA was reviewed by Assal et al. [15]. The NAT algorithm (Figure 1) developed for Indian ID-NAT users was employed which was similar to that described in Grabarczyk et al. [16].

### 2.5. Supplementary Assays

The NAT yield samples were retested for anti-HIV-1/anti-HIV-2, anti-HCV, and HBsAg by an alternative, more sensitive Chemiluminescent Immunoassay (ChLIA) (Architect Plus i1000 SR, Abbott Laboratories, Abbott Park, IL, USA) at an accredited laboratory in order to verify the screening results by ELISA. In order to detect the presence of any occult HBV infections (OBI), supplemental testing for anti-HBc, anti-HBs, and anti-HBe was performed by ChLIA (Architect Plus i1000 SR, Abbott Laboratories, Abbott Park, IL, USA). OBI is the presence of HBV DNA in blood or tissues without detectable HBsAg, with or without the presence of anti-HBc antibodies or anti-HBs antibodies, out with the preseroconversion window period [17]. Viral load of all NAT yield samples was also determined using real time quantitative PCR.

## 3. Results

A total of 213 (2.13%) donations (*N* = 10, 015) were found out to be reactive for either of the viral markers tested by either serology or NAT (both PUA and PUE). Overall prevalence of viral markers was 1.44% for HBV, 0.4% for HCV, 0.25% for HIV, and 5 (0.05%) coinfections (Table 1). Concordant serological and NAT reactive results were found in 153/213 (71.8%) reactive donations.

Thirty-nine cases (18.3%) were found to be serology reactive which when tested in duplicate were found to be consistent with the initial serological results and thus were labelled as “potential seroyields” (repeatedly seroreactive and NAT negative). These included 5 reactive samples for HBsAg, 22 for anti-HCV, and 12 for anti-HIV-1/anti-HIV-2.

Twenty-one (9.9%) samples were found to be initially reactive only by ID-NAT (PUA and/or PUE). Following the departmental protocol, all the initial reactive samples were found to be repeatedly reactive (RR) in ≥1 primary pilot tube and/or plasma bag when further tested in triplicate. Discriminatory assay discriminated all the RR samples (discriminated NAT yields) except for one which was found to be discriminatory nonreactive (DNR, namely, nondiscriminated NAT yields) (Table 2).

Of all the NAT yield cases, 3 were positive by both PUA and PUE assays and 18 were reactive only by PUE (15 HBV and 3 HCV yields). Seventeen HBV yields were occult infections and 1 was window period (WP yield) infection. All 3 HCV yields were window period infections. The results of supplemental test and quantitative viral loads for all the yield samples are depicted in Table 2. The overall NAT yield was found to be 1 in 477 and the virus-specific NAT yields were found to be 1 in 668 for HBV, 1 in 10,015 for HCV, and 1 in 2,003 for coinfections, respectively.

Of note, PUE assay detected 2 HBV cases which escaped detection by PUA but were HBsAg reactive (PUA miss). PUE also labelled 5 samples as coinfections (2 HIV-HBV, 3 HCV-HBV) which were identified as monoinfections in older PUA as well as serology. Three HBV and 2 HCV were flagged as coyields in PUE.

## 4. Discussion

The introduction of improved ID-NAT and serology tests measuring pathogen-specific humoral immune responses in the donor led to safer blood supply [18]. In Indian context, though NAT is not mandated, many blood centres have started NAT as an additional safety layer for better transfusion services [19]. Makroo et al., in their first Indian multicentric study, evaluated ID-NAT and found a NAT yield of 1 in 2622 donations and implicated that the routine NAT along with serological testing would significantly improve the blood safety in India [10]. Studies done previously at our institution with earlier versions of the assay reported yield of 1: 610 and 1: 628 which were higher than the previous studies [20, 21].

The virus-specific NAT yields for PUE were found to be 1 in 668 for HBV, 1 in 10,015 for HCV, and 1 in 2,003 for coinfections, respectively. However, virus-specific NAT yield for PUA was found to be 1 in 3,338 for HBV only. Hence, PUE system was evidently more efficient in detection of HBV and HCV. Tsoi et al. screened 517,072 and 399,326 consecutive donations for HBV by ID-NAT using Ultrio and Ultrio Plus assays, respectively, and reported enhanced detection of HBV after introduction of a more sensitive Ultrio Plus assay [22]. Vermeulen et al. reported similar results in their study on South African donor population [23]. Higher NAT yield in our study indicated the efficient detection of viral genome with the newer PUE system owing to its efficient target capture chemistry and better sensitivity to detect certain HBV genotypes (especially genotype D which is most prevalent in India) [16, 24].

In 9 out of 21 NAT yield cases, viral load could not be quantified. This could be attributed to the small amount of target present and sampling variability consistent with Poisson's distribution and also to the differences in sensitivities of the assay as there is large variation in limit of detection (LOD) for different strains and genotypes. We assessed the ability of ID-NAT to detect occult HBV infections by qualitative assessment of anti-HBc antibody on NAT yield cases. Out of combined NAT yield of 18 HBV cases, we detected 17 occult HBV infections and 1 window period donation which escaped detection by serology. Doda et al. also reported HBV NAT yield of 18 in which 12 cases were OBI and 6 WP (WP yield) donations [25].

We detected one WP HBV infection and 3 WP HCV infections (Table 2). HBV was detected with both assays whereas HCV infections were detected only by PUE. The results were in agreement with the claims of the manufacturer stating that newer PUE has enhanced capability of target capture along with increase in LOD in comparison to the PUA.

OBI is a disparate group of HBV-related conditions with a low level of circulating HBV DNA, and though the infectivity of such donors is a long-known fact, it is impractical to implement routine anti-HBc screening to curtail OBI transmission where the prevalence of anti-HBc is >10% [17]. Yugi et al. reviewed the HBV screening strategy and recommended that combining anti-HBc titer measurement along with HBV NAT would result in optimum blood safety in Japan (high prevalence zone for HBV) [26]. A study from Taiwan suggests that in HBV endemic regions the introduction of NAT in combination with HBsAg would provide extra safety to the blood transfusion without affecting donor pool [27]. Makroo et al. in their study measuring anti-HBc in blood donors also made a similar observation [28]. As 8–18% of donor population in India is anti-HBc reactive, inclusion of this marker for TTI screening would invariably result in attrition of large number of donors, causing shrinkage of the effective donor pool [29]. Our study suggests that, for endemic regions with high prevalence of HBV, inclusion of NAT to routine serology is a better alternative than and equally, if not more, effective to introducing universal anti-HBc screening.

Quantitative PCR failed to quantify the viral loads of the HCV specific NAT yield samples, namely, HCV WP infections (Table 2). The failure to quantify viral loads could be due to low levels of viremia and difference in the limits of the detection of either of the technologies. Although PUE and PUA have comparable detection limits for HCV, we could not find any possible explanation as to why the PUA missed these NAT yields (Table 3).

Both HIV-1 and HIV-2 coexist in India, HIV-1C being the commonest subtype reported [30]. HIV-2 cases among general and blood donor population have also been reported mostly from western and southern parts of India. As the threat of TTI is not static and is constantly evolving, the detection of HIV-2 may have serious repercussions on the blood supplies [31]. Although there is cross-reactivity between the main virus types (HIV-1/HIV-2), it is not sufficient to rely on an HIV-1 specific assay to detect all cases of HIV-2 [32]. The newer PUE assay incorporates HIV-2 detection which was not present in the previous versions. While writing this paper, no transfusion transmitted HIV-2 case has been found and documented at our centre or anywhere else in North India. However, it is only a matter of time for such a case to emerge [33].

Donor follow-up and look-back studies are the most important steps in verifying the TTI reactivity of the donors or the recipients [34]. Unlike most of the developed countries, Indian haemovigilance program and donor-vigilance are still in infancy. Routine donor follow-up is not possible as most of the donors do not turn up at transfusion facility upon receiving notifications of their TTI status [21]. Thus, it is difficult to ascertain the seroconversion in most of the donors and the transfused recipients as well. The strengthening of screening tests with incorporation of modern testing techniques and rigorous donor selection are the only remaining alternatives for developing nations to increase the blood safety.

## 5. Limitations

First, a confirmatory test was not performed and donors were notified only on the basis of repeat reactive screening results. Secondly, donor follow-up was not performed in our facility because the reactive donors were referred to respective departments where counselling, confirmatory testing, and management were done. (Unfortunately, results and data of reactive donors after referral from transfusion facility were not available with us.) Moreover, our study population was comprised of mostly replacement donors that may not reflect the situation for voluntary nonremunerated blood donor population.

## 6. Conclusion

Our study showed that the PUE has enhanced sensitivity (as measured in terms of NAT yields) compared to PUA for HBV and HCV. Since most of the yield cases were of HBV, introduction of PUE ID-NAT screening in India will reduce transmission of HBV. Full automation, reduced workspace, and infrastructure requirements with increased sensitivity and HIV-2 detection are additional advantages of the newer system.

## Figures and Tables

**Table 1:** NAT and serology results.

Total samples tested 10015						
Infectious marker	Serology and NAT concordant results	Seroyield	Total NAT yield	NAT yield		Total
				PUA	PUE	
HBV	124	5	15	3	15	144 (1.44%)
HCV	16	22	1	0	1	39 (0.4%)
HIV	13	12	0	0	0	25 (0.25%)
Coinfection	—	—	5^#^		5^#^	5 (0.05%)
Total	153	39	21	3	21	213 (2.14%)

^#^2 HIV/HBV coinfections (2 HBV cases detected only by NAT) and 3 HCV/HBV coinfections (1 HBV and 2 HCV cases detected only by NAT).

**Table 2:** Detailed serology results and viral load of NAT yields detected by both PUA and PUE assay.

Test summary, total samples tested 10015											
S. number	Sample ID	UE	Ultrio	Serology	Serology	Viral load	Supplemental serology (CMIA)			Interpretation	Remarks
				(ELISA)	(CMIA)	(IU/mL)	Anti-HBc total	Anti-HBs	Anti-HBe		
1	3301	HBV	NR	NR	NR	12	R	R	R	Occult HBV	PUE yield
2	3404	HBV	NR	NR	NR	ND	R	NR	R	Occult HBV	PUE yield
3	4004	HIV/**HBV**	HIV	Anti-HIV	Anti-HIV	HBV-ND	Equivocal^*∗*^	NR	R	Occult HBV	HBV/HIV coinfection (extra HBV detected by PUE only)
4	4278	HBV	NR	NR	NR	68	Equivocal^*∗*^	NR	R	Occult HBV	PUE yield
5	4737	HBV	NR	NR	NR	13	R	NR	NR	Occult HBV	PUE yield
6	6326	HBV	NR	NR	NR	105	R	NR	NR	Occult HBV	PUE yield
7	7750	HIV/**HBV**	HIV	Anti-HIV	Anti-HIV	HBV-35	R	NR	R	Occult HBV	HBV/HIV coinfection (extra HBV detected by PUE only)
8	4863	HBV	NR	NR	NR	ND	R	R	NR	Occult HBV	PUE yield
9	9020	HBV	NR	NR	NR	<10	R	NR	NR	Occult HBV	PUE yield
10	10267	HBV	NR	NR	NR	20	R	NR	NR	Occult HBV	PUE yield
11	4502	HBV/**HCV**	HBV	HBsAg	HBsAg	ND	NR			WP HCV infection	HBV/HCV coinfection (extra HCV detected by PUE only)
12	10187	HCV	NR	NR	NR	ND	NR			WP HCV infection	PUE yield
13	10489	HBV/**HCV**	HBV	HBsAg	HBsAg	ND	NR			WP HCV infection	HBV/HCV coinfection (extra HCV detected by PUE only)
14	30765	HBV	NR	NR	NR	ND	R	NR	R	Occult HBV	PUE yield
15	31956	HBV	NR	NR	NR	ND	R	NR	R	Occult HBV	PUE yield
16	31257	HBV	HBV	NR	NR	110	NR	NR	NR	WP HBV infection	PUA and PUE yield
17	31317	HBV	RR & DNR	NR	NR	35	R	NR	NR	Occult HBV	PUE yield
18	32029	HBV	HBV	NR	NR	30	R	NR	NR	Occult HBV	PUA and PUE yield
19	32112	HBV	HBV	NR	NR	26	R	R	NR	Occult HBV	PUA and PUE yield
20	30992	HBV	NR	NR	NR	12	R	NR	NR	Occult HBV	PUE yield
21	30589	HCV/**HBV**	HCV	HCV	HCV	HBV- ND	R	R	NR	Occult HBV	HBV/HCV coinfection (extra HBV detected by PUE only)

^*∗*^Equivocal (even after repeat testing in duplicate); R: reactive; NR: nonreactive; RR: repeat reactive; DNR: discriminatory nonreactive; ND: not detected, PUE: Procleix Ultrio Elite; PUA: Procleix Ultrio assay.

**Table 3:** Sensitivity of different nucleic acid testing assays used.

Viral marker	95% detection levels (95% fiducial limits)		
	Procleix Ultrio assay [11]	Procleix Ultrio Elite assay [12]	Quantitative PCR (equipment used)
HIV-1	20.72 copies/mL	18.0 IU/mL	NA
HIV-2	NA	10.4 IU/mL	Not tested
HCV	2.78 IU/mL	3.0 IU/mL	18 IU/mL (Roche Cobas TaqMan Test) [13]
HBV	7.46 IU/mL	4.3 IU/mL	6.40 IU/mL (Abbott real time PCR m2000) [14]
